# Surgery

**Published:** 1896-06

**Authors:** Jane W. Carroll

**Affiliations:** Buffalo, N. Y.


### Progress in Medical Science.

### SURGERY.

Conducted by JANE W. CARROLL, M. D., Buffalo, N. Y.

### EARLY MANAGEMENT OF CONGENITAL CLUBFOOT.

LEWIS A. SAYRE (*Medical Record*, April 11, 1896,) enters a strong protest against the prevalent practice of "leaving the child until old enough to stand it." He considers that the treatment should be commenced before the child is forty-eight hours old, and twenty-four is better still.

The method for procedure is for the operator to take the foot in his two hands and gradually, but very gently, press it around toward the normal position. As this is done you will observe the toes and front part of the foot will become blanched as white as snow and apparently perfectly bloodless. If retained in this position too long, of course sloughing would necessarily follow this obstruction to the circulation. Therefore, after holding the foot in this improved position for a few seconds only, you relax your hold upon the foot, which will immediately recede to its former deformed position, and as it does so you will observe the gradual return of the pink color and the natural circulation to the foot and toes.

If both feet are deformed, as is generally the case, take the other and treat it in the same manner.

Repeat the manœuver a number of times and instruct the nurse how to do the same, and see that it is done every few hours day and night. If these manœuvers are not sufficient, the feet may be treated with the usual dressing of plaster-of-Paris. Dr. Sayre concludes by emphasising (1) the necessity of commencing the treatment at birth ; (2) that there is no instrument to be compared with the human hand in rectifying the deformity ; (3) no means of retaining the parts in position equal to plaster-of-Paris properly applied.

---

### SURGICAL TREATMENT OF MALIGNANT DISEASE OF THE BREAST.

Sanderson, of the Women's Hospital, Brighton, (*Lancet*, December 21, 1895,) claims that the whole affected mamma should be freely and thoroughly excised.

As a matter of fact the efferent lymphatic vessels run from the axillary glands through the apex of the axilla to the posterior triangle and, after forming connections with the glands therein, finally enter the right thoracic duct on the right and the left on the left side. Moreover, superficial lymphatics from the skin covering the mamma track up over the clavicle to these same glands in the posterior triangle. It is laid down, says the author, that in primary cancer of the breast the axilla should be cleared whether the glands are visibly affected or not. Why ? Because the fact that they are not affected macroscopically is no proof that they are not affected microscopically ; and yet, in the face of these anatomical facts, it is not the usual custom or practice to go further.

It is wrong to presume that the axilla is free because it shows no sign to the naked eye or the finger ; it is equally wrong to assume that the posterior triangle has escaped.

Still more is it inconceivable how the triangle can be left if the axillary glands are found affected at the time of the operation.

It may be objected that it is not feasible or is difficult to clear the posterior triangle. It may be done a week or ten days after the primary operation.

A flap formed by a long incision down the sterno-mastoid muscle, meeting an incision along the clavicle, is detached as far back as the anterior border of the trapezius ; this exposes the triangle and the contents can be removed with a little care and patience. It is important to remember that lymphatic glands may be affected by carcinoma, directly or indirectly. In carcinoma of the breast the lymphatic glands most frequently affected are those of the axilla, especially the pectoral set lying under the border of the pectoralis minor. Later in the case, glands higher up, situated in the posterior triangle, become also involved.

It is extremely rare, says the writer, for the glands above the clavicle to be affected directly. In any case to reduce the chance of a recurrence to a minimum, the glands of the axilla should be cleared, even if no enlarged glands are felt.

If none are found, the triangle has probably escaped and it is undesirable to subject the patient to increased risk by extending the operation. But if the axilla is markedly involved, there is great probability that the triangle is also, and there is no reasonable chance of cure unless it is also cleaned out.

The superficial glands, lying in the cervical fascia, can be readily taken away, but it is no easy matter to remove the deeper glands which lie around the subclavian vessels and under cover of the clavicle, and these deeper glands are the ones most likely to be affected. Yet he says it is undoubtedly advisable to attempt their removal in many cases where they are now left untouched.—*New York Medical Journal.*

---

### THE MICROSCOPE IN SURGERY.

SENN, in his recent work on tumors, (*Medical Review,*) states the microscope is not so serviceable in diagnosticating tumors as many suppose, and cites as an instance the case of the late Emperor Frederick of Germany. Small pieces of tumor or scrapings of tissue should not be sent to the pathologist simply to see what the microscope will reveal or what the pathologist knows. The object is to obtain a correct diagnosis, and, to this end, as large a piece of tumor as possible should be sent for examination. It should be accompanied with a history of the case and all other points, such as site, character of growth, and the like. In this way the microscope usually decides when the appearance to the naked eye throws doubt on the character of the tumor.

---

### A NEW METHOD IN THIGH AMPUTATIONS.

DAWBARN, (*Med. Record*), of the polyclinic, presented to the Academy of Medicine, December, 1895, a new method in thigh amputations, although the same had been taught by him for the past ten years, namely : hamstringing the patient just prior to any thigh amputation. "The technique is as follows : The thigh being elevated and 'milked' as usual and the leg held extended on the thigh, thereby causing the hamstring tendons to stand out tensely, the surgeon divides these on both sides with two bold strokes of the knife," bleeding being arrested by the thumb of an assistant.

The Esmarch bandage is now applied above the point at which it is decided to amputate. The reason for hamstringing is obvious. None of the hamstring muscles, save part of one, the biceps, has any attachment to the thigh bone, but all arise from the pelvis and are inserted below the knee. These are the sartorius, gracilis, semi-tendinosus and semi-membranosus. Also the biceps, except as aforesaid. Consequently, when the muscles are cut across in thigh amputations nothing prevents their retraction for a long distance. All the other muscles of the thigh, as the vastus externus, vastus internus, crureus and the adductors, are attached to the thigh bone, and for a considerable distance. Consequently, when cut through in thigh amputation they cannot retract far. Every surgeon knows practically that the thigh muscles do retract to very unequal distances in the stump. For this reason the "suture en etage" was devised, sewing together the muscles in the interior of the stump tier after tier to make a comparatively solid muscular end instead of one full of holes or dead spaces.

In hamstringing the patient, therefore, those muscles at once shorten up, and when a little later we proceed to amputate the thigh we shall find that our stump end will be quite smooth, for all the muscles will retract about equally.

The same principle may be applied in other amputations, as at the tendon Achilles in leg amputations, permitting the gastrocnemius to shorten, and the biceps humero-tendon before amputation through the upper arm.

---

### PICRIC ACID FOR BURNS.

French surgeons (*Medical Times and Hospital Gazette*) have recently been using a solution of picric acid for the first treatment of burns. The pain can be almost immediately alleviated or removed by painting the affected surface with strong solution of picric acid.

The remedy has proved harmless, and the yellow stain can be easily washed out with boric acid.

The general verdict has been that this remedy has greatly lessened suffering and therefore probably saved life, and even severe cases thus treated have recovered more speedily and completely than would have been the case under any other form of treatment. It would be well, therefore, for medical officers of factories or workshops where accidents from burning or scalding are common occurrences to try the experiment and direct that solutions of the acid should be kept on hand and explain the method of application to the workers.

